# Eicosapentaenoic acid ameliorates hyperglycemia in high-fat diet-sensitive diabetes mice in conjunction with restoration of hypoadiponectinemia

**DOI:** 10.1038/nutd.2016.21

**Published:** 2016-06-27

**Authors:** M Morimoto, E-Y Lee, X Zhang, Y Inaba, H Inoue, M Ogawa, T Shirasawa, O Yokosuka, T Miki

**Affiliations:** 1 Department of Medical Physiology, Chiba University, Graduate School of Medicine, Chiba, Japan; 2 Department of Gastroenterology and Nephrology, Chiba University, Graduate School of Medicine, Chiba, Japan; 3 Metabolism and Nutrition Research Unit, Innovative Integrated Bio-Research Core, Institute for Frontier Science Initiative, Kanazawa University, Kanazawa, Japan; 4 Department of Neurology, University of Michigan, Ann Arbor, MI, USA

## Abstract

**Background/Objective:**

Eicosapentaenoic acid (EPA) exerts pleiotropic effects on metabolic disorders such as atherosclerosis and dyslipidemia, but its effectiveness in the treatment of type 2 diabetes mellitus remains controversial.

**Methods:**

We examined the antidiabetic effect of EPA in insulin receptor mutant (*Insr*^*P1195L/+*^) mice that exhibit high-fat diet (HFD)-dependent hyperglycemia.

**Results:**

EPA supplementation was found to alleviate hyperglycemia of *Insr*^*P1195L/+*^ mice fed HFD (*Insr*^*P1195L/+*^/HFD mice), which was accompanied by amelioration of increased gluconeogenesis and impaired insulin signaling, as assessed by glucose-6-phosphatase (*G6pc*) expression on refeeding and insulin-induced phosphorylation of Akt in the liver, respectively. We found that serum levels of adiponectin, the antidiabetic adipokine, were decreased by HFD along with the body weight gain in *Insr*^*P1195L/+*^ mice but not in wild-type mice, suggesting that *Insr*^*P1195L/+*^ mice are prone to hypoadiponectinemia in response to obesity. Interestingly, the blood glucose levels of *Insr*^*P1195L/+*^ mice were in reverse proportion to their serum adiponectin levels and EPA supplementation ameliorated their hyperglycemia in conjunction with the restoration of hypoadiponectinemia.

**Conclusions:**

EPA exerts an antidiabetic effect in *Insr*^*P1195L/+*^/HFD mice, an HFD-sensitive, insulin-resistant animal model, possibly through its action against hypoadiponectinemia.

## Introduction

Type 2 diabetes mellitus (T2DM) is a metabolic disorder characterized by chronic hyperglycemia, and the number of patients with T2DM has been increasing worldwide. Many environmental factors such as overnutrition and sedentary lifestyle are considered to be responsible for this increase.^1^ Especially, excessive intake of fat rich in saturated fatty acid is considered to be associated with the development of insulin resistance and consequential T2DM.^2^ Recently, we found that high-fat diet (HFD) feeding of a mouse harboring a loss-of-function mutation in the insulin receptor (*Insr*^*P1195L/+*^) developed overt hyperglycemia.^3^ This mutation is a single amino-acid substitution from proline to leucine at 1195 amino-acid residue (P1195L), which acts as a dominant-negative mutant in the heterozygote. In *Insr*^*P1195L/+*^ mice fed HFD (*Insr*^*P1195L/+*^/HFD mice), lipolysis in white adipose tissue (WAT) and gluconeogenesis in the liver were both increased, and these are considered to be responsible for hyperglycemia. Lipolysis of triglyceride (TG) generates fatty acids and glycerol. Fatty acids are classified into three groups: saturated, monounsaturated, and polyunsaturated (PUFA) fatty acids. PUFA is further subclassified into *n*-6 PUFA and *n*-3 PUFA. The latter has a double bond at the third carbon from the methyl end of the carbon chain. The HFD used in our study contains a high percentage of saturated fatty acid, which is known to induce chronic inflammation in WAT, worsening insulin resistance.^4^ By contrast, *n*-3 PUFAs such as eicosapentaenoic acid (EPA) and docosahexaenoic acid have been reported to suppress chronic inflammation through various pathways involving Toll-like receptor 4, resolvin E1 and GRP120.^5, 6, 7^

In the present study, we hypothesized that chronic inflammation in WAT might contribute to the development of hyperglycemia in *Insr*^*P1195L/+*^/HFD mice. To test this, we administered EPA to *Insr*^*P1195L/+*^/HFD mice and evaluated chronic inflammation and glucose metabolism in these mice. Although chronic inflammation was not increased in *Insr*^*P1195L/+*^/HFD mice compared with that in wild-type (WT)/HFD mice, EPA supplementation significantly ameliorated hyperglycemia of *Insr*^*P1195L/+*^/HFD mice. In the present study, we examined the underlying mechanism for improved hyperglycemia in *Insr*^*P1195L/+*^/HFD mice by EPA.

## Materials and methods

### Animals

**Figure 1:**
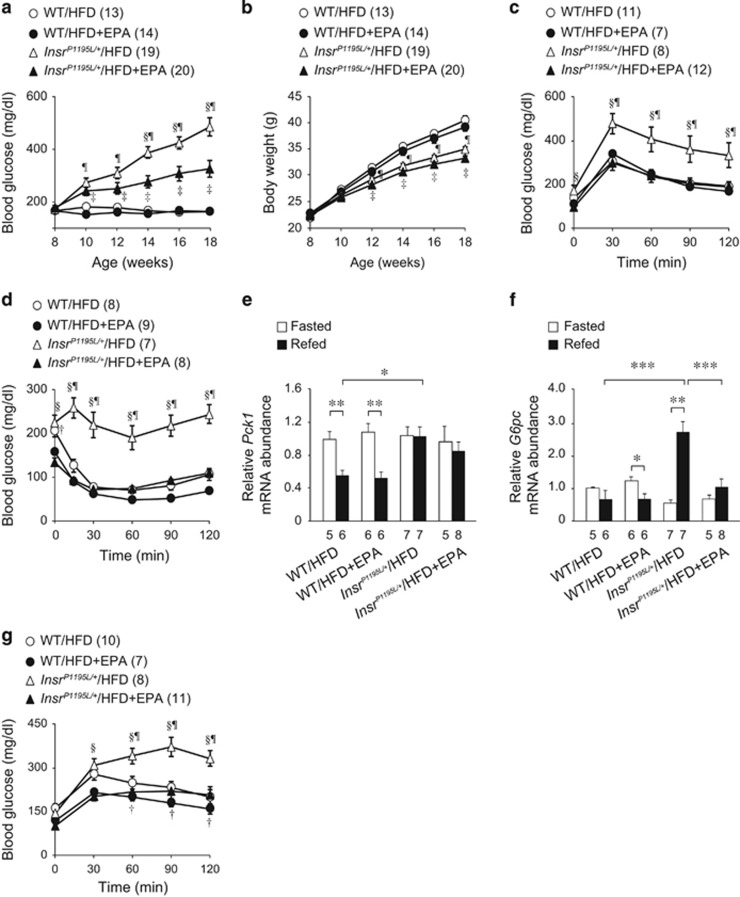
Amelioration of glucose metabolism by EPA supplementation. (**a**) Changes in blood glucose levels fed *ad libitum*. (**b**) Changes in body weight. (**c**) Blood glucose levels after oral glucose loading (1 g kg^−1^). (**d**) Blood glucose levels after intraperitoneal insulin administration (0.75 U kg^−1^). (**e**, **f**) mRNA expression levels of *Pck1* (**e**) and *G6pc* (**f**) in the liver at fasted (after 16 h fasting) and refed (after 3 h refeeding) state. *P<0.05, **P<0.01, ***P<0.001. (**g**) Changes in blood glucose levels after intraperitoneal glycerol administration (0.5 g kg^−1^). Data are mean±s.e.m. (**a**–**d**, **g**) ^¶^*P*<0.05 (*Insr*^*P1195L/+*^/HFD vs WT/HFD); ^§^*P*<0.05 (*Insr*^*P1195L/+*^/HFD vs *Insr*^*P1195L/+*^/HFD+EPA); ^†^*P*<0.05 (WT/HFD vs WT/HFD+EPA); ^‡^*P*<0.05 (*Insr*^*P1195L/+*^/HFD+EPA vs WT/HFD+EPA). The number in the parenthesis (**a**–**d**, **g**) and in the bottom of the columns (**e**, **f**) denotes sample size.

Mice were allowed free access to water and laboratory chow. All mouse experiments except for Figures 1a and b were performed in male mice aged 16–18 weeks. They were kept under 12-h light/12-h dark cycle. For normal diet (ND) feeding and for feeding before the initiation of HFD, mice were maintained on standard chow (CE-2) (12.1% kcal from fat; Clea Japan Inc., Tokyo, Japan). *Insr*^*P1195L/+*^/HFD and WT/HFD mice with C57BL/6 background were raised as described previously.^3^ Briefly, mice were maintained on a HFD (D12492) (60.0% kcal from fat) (Research Diets Inc., New Brunswick, NJ, USA) starting at 8 weeks of age unless stated otherwise. For EPA supplementation, EPA (kindly provided by Mochida Pharmaceutical Co. Ltd., Tokyo, Japan) was added to the HFD (5% *wt/wt*) and was given to the mice starting at 8 weeks of age. Each type of chow was assigned randomly. Animal care and experiments were performed in accordance with the guidelines of Chiba University, Japan. No sample size estimation and blinding was carried out in the experiments.

### Metabolic analysis and blood biochemistry

Glucose tolerance test (oral glucose tolerance test), insulin tolerance test and glycerol tolerance test were performed as previously described.^8^ Briefly, for oral glucose tolerance test, the mice were fasted for 16 h and glucose (1 g kg^−1^) was administered orally. For insulin tolerance test and glycerol tolerance test, 16-h-fasted mice were administered intraperitoneally with 0.75 IU kg^−1^ insulin and 0.5 g kg^−1^ glycerol, respectively. Blood glucose was measured as previously described.^8^ Serum concentrations of total adiponectin (Otsuka pharmaceuticals, Tokyo, Japan) and free fatty acid (FFA) (BioVision, Inc., San Francisco, CA, USA) were measured using the commercially available kits. TG content in the liver was measured using the kit from BioVision, Inc.

### Primers and quantitative reverse transcriptase-PCR

Total RNA was isolated from the WAT (epididymal fat pad) and liver and subjected to quantitative reverse transcriptase-PCR analyses using SYBR Green (Thermo Fisher Scientific, Waltham, MA, USA), according to the manufacturer's protocol. The primers used were 5′-CACCATCACCTCCTGGAAGA-3′ and 5′-GGGTGCAGAATCTCGAGTTG-3′ for *Pck1*, 5′-GTGGCAGTGGTCGGAGACT-3′ and 5′-ACGGGCGTTGTCCAAAC-3′ for *G6pc*, 5′-CGGAGTCCGGGCGGT-3′ and 5′-GCTGGGTAGAGAATGGATGAACA-3′ for *Tnfa*, 5′-CTTTGGCTATGGGCTTCCAGTC-3′ and 5′-GCAAGGAGGACAGAGTTTATCGTG-3′ for *Emr1*, 5′-AGAGATGGCACTCCTGGAGAGAA-3′ and 5′- CAACATCTCCTGTCTCACCCTTA-3′ for *Acrp30*, 5′-GTTTTATGCTGTTATGGGTG-3′ and 5′-GTAATTTCTTGTGAAGTGCTCATAG-3′ for *Pparg2* 5′-TACAGAGTGCTGGCCAAGAG-3′ and 5′-GCGTCGTGATTAGCGATGA-3′ and 5′-ATGGCCTCCCATCTCCTT-3′ for *Hprt*.

### Western blotting

Akt phosphorylation in the liver was examined in the four mouse groups (*Insr*^*P1195L/+*^ and WT mice under either HFD or HFD+EPA) at 18 weeks of age (after 10 weeks of HFD feeding). The cervical vein was exposed and 0.1 IU kg^−1^ insulin was injected via the vein. Five minutes later, the liver was removed. Tissues were lysed in sonication buffer (20 mm HEPES pH7.5; 150 mm NaCl; 25 mm EDTA; 1% NP-40; 10% glycerol, 1 mm sodium vanadate, 1 mm phenylmethylsulfonyl fluoride and protease and phosphatase inhibitors). Fifty micrograms of protein was subjected to sodium dodecyl sulfate-polyacrylamide gel electrophoresis. Antibodies against the following proteins were used (all from Cell Signaling Technology, Danvers, MA, USA): phospho-Akt (p-S473) (1:1000, no. 9271), Akt (1:1000, no. 9272), and β-actin (1:1000, no. 4967). Band intensities were quantified by the Image J Software (National Institutes of Health, Bethesda, MD, USA).

### Primary hepatocyte experiments

Primary mouse hepatocytes were isolated as previously described.^9^ Briefly, the livers of 18-week-old male mice were perfused through the portal vein (5 ml min^−1^ for 17 min at 37 °C) with 1 × HBSS (WAKO, Osaka, Japan) supplemented with 10 mm HEPES (GIBCO, Grand Island, NY, USA), 0.4 mg ml^−1^ type 1 collagenase (CLS-1, Worthington, Lakewood, NJ, USA) and protease inhibitor cocktail (cOmplete, EDTA-free, Roche, Basel, Switzerland). After filtration and washing, hepatocytes were isolated using a 45% (*v*/*v*) Percoll (GE Healthcare, Buckinghamshire, UK) solution. The hepatocytes were resuspended in William's E medium (Sigma-Aldrich Co., St Louis, MO, USA) containing 10% fetal bovine serum, 100 mm dexamethazone, 10 nm insulin and 1 × Penicillin–Streptomycin–l-Glutamine Solution (WAKO), plated to six-well plate (5.0 × 10^5^ hepatocytes per well) and cultured for 10 h. The medium was then changed to William's E medium containing 1% bovine serum albumin with or without 100 nm EPA sodium salt (Sigma-Aldrich) and/or 5 μm LY249002 (Sigma-Aldrich), and the cells were cultured for 24 h. Hepatocytes were stimulated with or without 10 nm insulin for 5 min, harvested and sonicated. Thirty micrograms of protein was subjected to western blotting.

### Histological examination

Histological analyses were performed as previously described.^10^ WAT and liver were dissected and fixed in 10% buffered formalin overnight and embedded in paraffin. Sections (4 μm) were stained with hematoxylin and eosin (HE) for histological analysis. For immunofluorescence staining of caveolin, sections were incubated in 1.5% goat serum dissolved in PBS-T (phosphate-buffered saline containing 0.3% Tween-20) for 1 h at room temperature. Sections were stained with rabbit anti-caveolin antibody (1:500, no. 610060, BD Biosciences, Franklin Lakes, NJ, USA) overnight at 4 °C. Subsequently, incubation with secondary antibody (donkey anti-rabbit IgG antibody conjugated with Alexa-Fluor 555, 1:1000, Thermo Fisher Scientific) was performed for 90 min at room temperature. For imaging, slides were examined under a Biozero fluorescence microscope (BZ-8100, Kyence, Osaka, Japan).

### Statistical analyses

Results are expressed as means±s.e.m. Data were analyzed using the statistical software package, IBM SPSS Version 19 (IBM Corporation, Armonk, NY, USA). After confirming all variables to be normally distributed, differences between two groups were assessed using the unpaired two-tailed Student's *t*-test. Data sets involving more than three groups were assessed by one-way analysis of variance with Bonferroni *post-hoc* test. *P*<0.05 was considered to be statistically significant.

## Results

### EPA ameliorated hyperglycemia in *Insr*^*P1195L/+*^ mice fed HFD

*Insr*^*P1195L/+*^ mice under HFD (*Insr*^*P1195L/+*^/HFD mice) developed hyperglycemia as previously reported.^3^ However, EPA supplementation significantly improved hyperglycemia of *Insr*^*P1195L/+*^/HFD mice from 14 to 18 weeks of age (Figure 1a). In addition, EPA-supplemented-*Insr*^*P1195L/+*^/HFD mice (*Insr*^*P1195L/+*^/HFD+EPA mice) exhibited modestly reduced body weight gain compared with *Insr*^*P1195L/+*^/HFD mice (Figure 1b). By contrast, EPA supplementation failed to evoke significant decrease in either body weight gain or blood glucose levels in WT mice. Glucose tolerance in *Insr*^*P1195L/+*^/HFD mice was markedly impaired (Figure 1c). However, EPA supplementation significantly attenuated the blood glucose rise after oral glucose loading. In addition, EPA supplementation improved insulin sensitivity in *Insr*^*P1195L/+*^/HFD mice, as assessed by insulin tolerance test (Figure 1d). The reduction in blood glucose levels of *Insr*^*P1195L/+*^/HFD mice by EPA supplementation became statistically insignificant at or later than 24 weeks of age (data not shown). Therefore, all the experiments were performed in the mice aged 16–18 weeks, unless otherwise specified.

We then examined whether EPA suppresses gluconeogenesis by quantifying the expression of gluconeogenesis-related genes, such as phosphoenolpyruvate carboxykinase 1 (*Pck1*) and glucose-6-phosphatase (*G6pc*). EPA supplementation did not affect *Pck1* expression in both WT/HFD and *Insr*^*P1195L/+*^/HFD mice (Figure 1e). *G6pc* expression in *Insr*^*P1195L/+*^/HFD mice was markedly increased after 3 h refeeding (Figure 1f), as previously reported,^3^ while EPA supplementation significantly suppressed *G6pc* expression in *Insr*^*P1195L/+*^/HFD mice. *G6pc* is a key enzyme involved in gluconeogenesis from glycerol as well as from amino acids and pyruvate. We therefore examined gluconeogenesis by measuring the blood glucose rise after intraperitoneal glycerol administration (Figure 1g). Although gluconeogenesis from glycerol was increased in *Insr*^*P1195L/+*^/HFD mice, as previously reported,^3^ the blood glucose rise after glycerol administration was significantly attenuated in *Insr*^*P1195L/+*^/HFD+EPA mice, suggesting that EPA supplementation may ameliorate hyperglycemia through suppression of *G6pc* expression.

### Akt phosphorylation by insulin was improved by EPA supplementation in *Insr*^*P1195L/+*^/HFD mice

**Figure 2:**
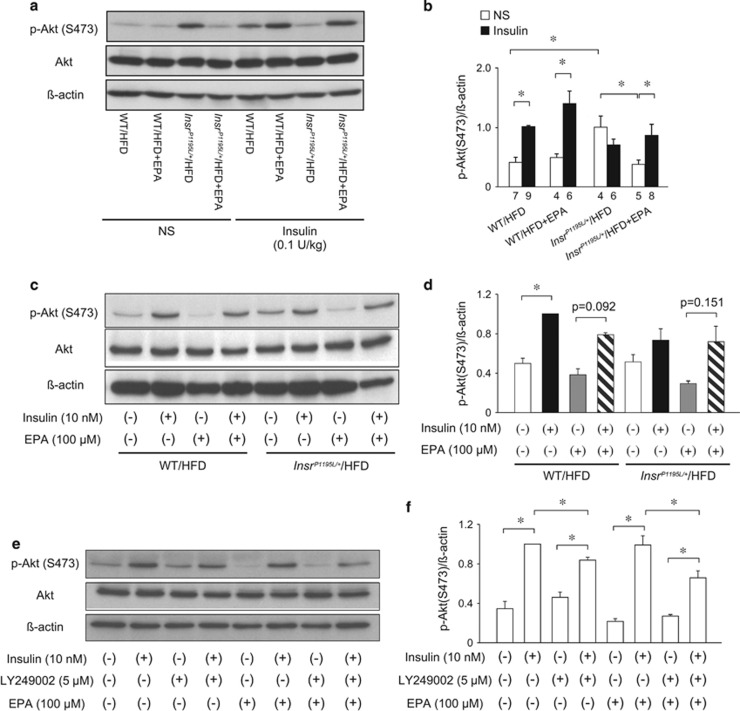
Phosphorylation of Akt in the liver and primary hepatocytes. (**a**, **b**) Akt phosphorylation after insulin administration (0.1 U kg^−1^, intravenous) *in vivo*. (**c**–**f**) Akt phosphorylation in primary hepatocytes. (**a**, **c**, **e**) A representative result of p-Akt. (**b**) Quantified result of p-Akt levels. The number in the bottom of the columns denotes sample size. (**d**, **f**) Quantified result of p-Akt levels (*n*=3 per each group). Data are mean±s.e.m. *P<0.05.

Insulin is known to inhibit gluconeogenesis in hepatocytes. As gluconeogenesis has been shown to be increased in *Insr*^*P1195L/+*^/HFD mice, we first evaluated insulin signaling in the liver *in vivo* by examining Akt phosphorylation after insulin administration (0.1 U kg^−1^) to jugular vein (Figures 2a and b). Quantification of phosphorylated Akt (p-Akt) revealed that insulin-induced Akt phosphorylation was significantly impaired in *Insr*^*P1195L/+*^/HFD mice compared with that in WT/HFD, as previously described.^3^ In WT/HFD mice, EPA supplementation did not affect insulin-induced Akt phosphorylation. Although insulin-induced Akt phosphorylation was blunted in *Insr*^*P1195L/+*^/HFD mice, EPA supplementation suppressed the basal p-Akt levels and was considered to restore the responsiveness of Akt phosphorylation to insulin in these mice.

We next examined whether EPA directly activates the insulin signaling cascade in primary hepatocytes. For this purpose, primary hepatocytes were isolated from *Insr*^*P1195L/+*^/HFD and WT/HFD mice, precultured in the presence or absence of EPA and subjected to stimulation with insulin (Figures 2c and d). In the absence of EPA, insulin significantly stimulated Akt phosphorylation in WT/HFD hepatocytes but not in *Insr*^*P1195L/+*^/HFD hepatocytes. EPA treatment partially ameliorated the insulin responsiveness in *Insr*^*P1195L/+*^/HFD hepatocytes, which resembles the trend of p-Akt by insulin administration *in vivo* (Figures 2a and b).

In search for the mechanism of EPA action on Akt phosphorylation, the primary hepatocytes of WT mice under ND (WT/ND mice) were pretreated with or without a phosphoinositide 3-kinase (PI3K) inhibitor LY294002 and insulin-induced Akt phosphorylation was examined (Figures 2e and f). EPA treatment did not affect insulin-induced Akt phosphorylation but rather tended to decrease its basal level. On the contrary, LY294002 suppressed the insulin-induced Akt phosphorylation similarly in the EPA-treated and EPA-untreated hepatocytes. Thus EPA supplementation *in vivo* may recapitulate the defective insulin-induced Akt phosphorylation in the *Insr*^*P1195L/+*^/HFD liver (Figure 2b) by lowering the basal p-Akt level through a PI3K-independent pathway.

### EPA modestly suppressed WAT inflammation of *Insr*^*P1195L/+*^/HFD mice

**Figure 3:**
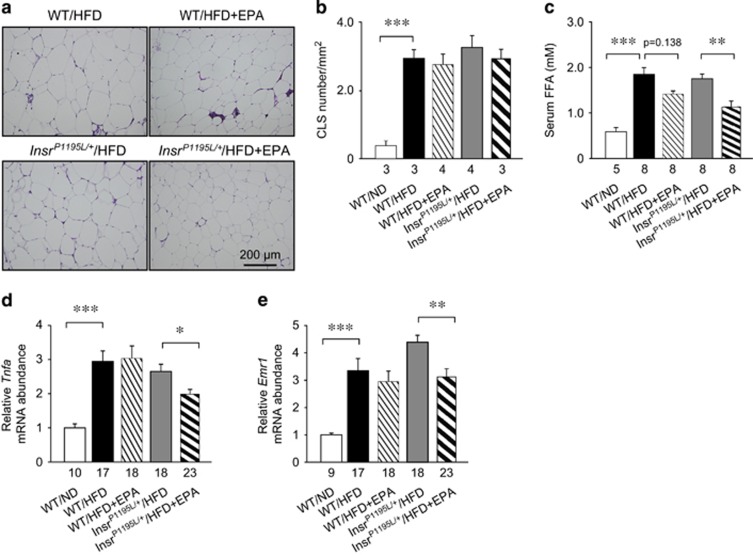
Chronic inflammation in WAT of *Insr*^*P1195L/+*^/HFD mice. (**a**, **b**) HE staining of epididymal fat. (**a**) A representative result. Scale bar: 200 μm. (**b**) CLS number. (**c**) Serum FFA levels. (**d**, **e**) mRNA expression levels of *Tnfa* (**d**) and *Emr1* (**e**). Data are mean±s.e.m. *P<0.05, **P<0.01, ***P<0.001. (**b**–**e**) The number in the bottom of the columns denotes sample size.

As EPA is well known to improve insulin sensitivity via its anti-inflammatory action in WAT,^11^ we examined the inflammatory changes in WAT of *Insr*^*P1195L/+*^/HFD+EPA mice. Chronic inflammation in WAT evokes morphological changes called crown-like structures (CLS), which are composed of macrophages surrounding dying or dead adipocytes.^12^ Histological examination by HE staining in WAT revealed that the number of CLS was significantly increased by HFD feeding (Figures 3a and b). However, the frequency of CLS was not different between *Insr*^*P1195L/+*^/HFD and WT/HFD mice and was not decreased by EPA supplementation.

We measured the serum FFA, a surrogate marker of lipolysis and inflammation^4^ and found that they were significantly increased by HFD (Figure 3c). There was no significant difference in FFA levels between *Insr*^*P1195L/+*^/HFD and WT/HFD mice, and EPA supplementation significantly lowered FFA in *Insr*^*P1195L/+*^/HFD mice.

We also examined the change in the mRNA expression levels of two inflammatory-related genes; *Tnfa* and *Emr1*, which encode tumor necrosis factor α and EGF (epidermal growth factor)-like module-containing mucin-like hormone receptor-like 1 (also known as F4/80), respectively (Figures 3d and e). HFD feeding of WT mice significantly increased the expression levels of these genes, but there was no significant difference between *Insr*^*P1195L/+*^/HFD and WT/HFD mice. By contrast, EPA supplementation significantly suppressed these expression levels in *Insr*^*P1195L/+*^/HFD mice. Accordingly, EPA supplementation may ameliorate the hyperglycemia of *Insr*^*P1195L/+*^/HFD mice, at least in part, by attenuating chronic inflammation.

### EPA supplementation reduced adipocyte size in WAT and adiposity *in vivo*

**Figure 4:**
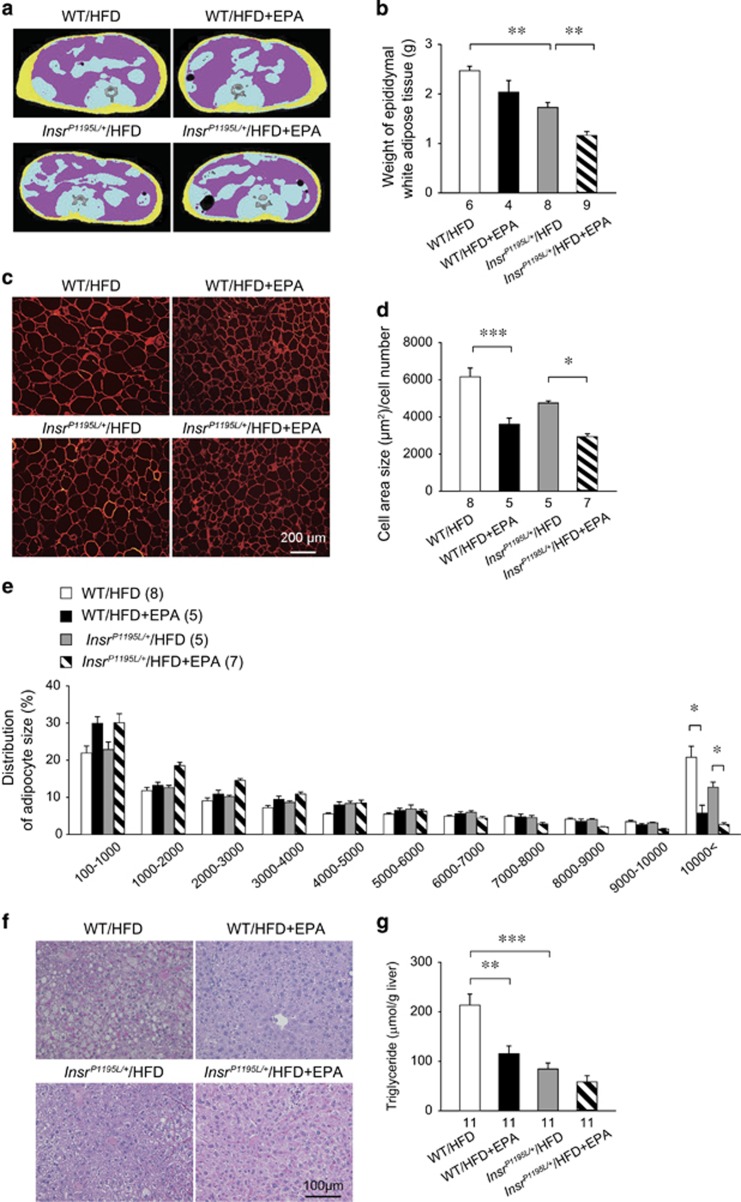
Effect of EPA supplementation on adiposity and steatosis in *Insr*^*P1195L/+*^/HFD mice at 18–20 weeks of age. (**a**) Computed tomographic images at the level of lower end of right kidney. Visceral and subcutaneous fat are indicated in pink and yellow, respectively. (**b**) Weight of epididymal WAT. (**c**) Immunostaining of caveolin in epididymal fat. (**d**) Mean adipocyte size. Scale bar: 200 μm. (**e**) Histogram of the cell area of four mouse groups. (**f**) HE staining of the liver. (**g**) TG content in the liver. (**b**, **d**, **e**, **g**) Data are mean±s.e.m. *P<0.05, **P<0.01, ***P<0.001. The number in the bottom of the columns (**b**, **d**, **g**) and in the parenthesis (**e**) denotes sample size.

HE staining revealed that WAT in EPA-supplemented mice contained a number of small adipocytes in both WT/HFD and *Insr*^*P1195L/+*^/HFD mice. We therefore performed computed tomography scanning of the four mouse groups (Figure 4a). As previously reported,^3^ the visceral fat area in *Insr*^*P1195L/+*^/HFD mice was reduced when compared with that in WT/HFD mice. In addition, computed tomography scanning suggested that adiposity might be reduced mildly by EPA supplementation. We also measured epididymal fat weight and found that it was significantly decreased by EPA supplementation in *Insr*^*P1195L/+*^/HFD mice (Figure 4b).

We then quantified adipocyte size by immunostaining of caveolin in WAT (Figures 4c–e). The average adipocyte size in *Insr*^*P1195L/+*^/HFD mice tended to be smaller than that in WT/HFD mice (*P*=0.10; Figure 4d), and EPA supplementation significantly reduced the average adipocyte size in both WT/HFD and *Insr*^*P1195L/+*^/HFD mice. Notably, EPA supplementation decreased the number of large (>10 000 μm^2^) adipocytes in both WT and *Insr*^*P1195L/+*^ mice (Figure 4e). In addition to its effect on WAT, EPA supplementation ameliorated the steatosis in the liver of WT/HFD mice as assessed by HE staining (Figure 4f) and TG content in the liver (Figure 4g). In *Insr*^*P1195L/+*^/HFD mice, the TG content was much lower than that in WT/HFD mice, and there was no further decrease by EPA supplementation.

### *Insr*^*P1195L/+*^ mice were susceptible to body weight-dependent reduction in serum adiponectin and EPA supplementation restored the reduction

**Figure 5:**
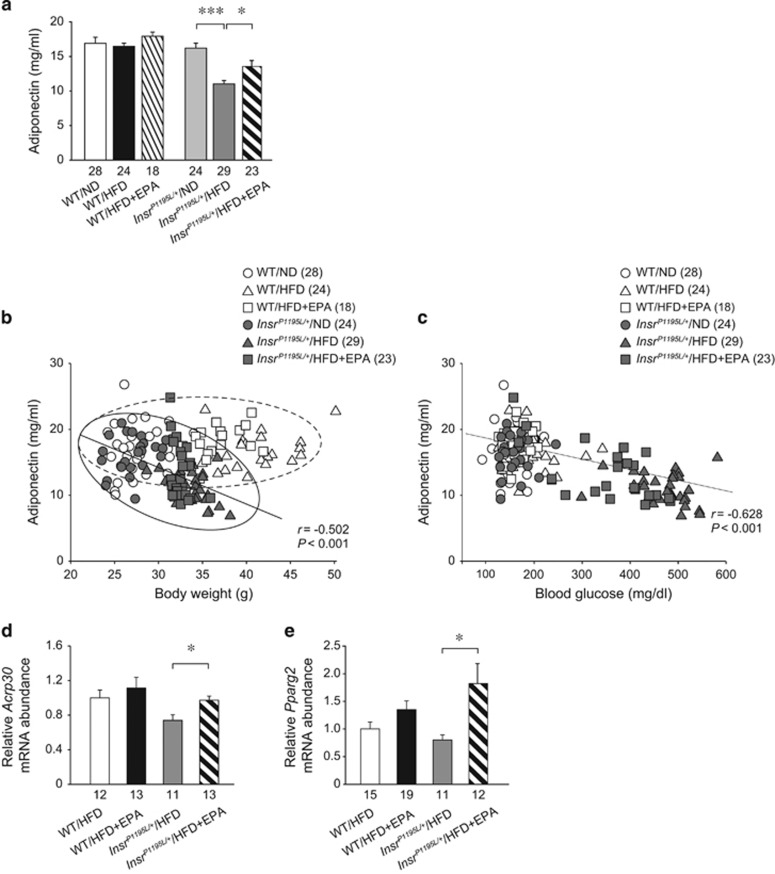
Serum adiponectin levels and their correlation with body weight or blood glucose levels. (**a**) Serum adiponectin levels fed *ad libitum* (16–18 weeks of age). (**b**) Relationship between body weight and adiponectin levels. Most of the data of *Insr*^*P1195L/+*^ mice and WT mice are plotted inside the solid- and dotted-circle, respectively. (**c**) Relationship between blood glucose levels and adiponectin levels. (**b**, **c**) *r*=Pearson's correlation coefficient. (**d**,** e**) mRNA expression levels of *Acrp30* (**d**) and *Pparg2* (**e**) in WAT under refed condition. Data are mean±s.e.m. *P<0.05, ***P<0.001. The number in the bottom of the columns (**a**, **d**, **e**) and in the parenthesis (**b**, **c**) denotes sample size.

As adiposity is known to be negatively correlated with serum adiponectin levels in humans and rodents,^13^ we examined serum adiponectin levels (Figure 5a). In this analysis, we also included the data of WT/ND and *Insr*^*P1195L/+*^/ND mice. The serum adiponectin levels in WT/HFD mice were not different from those in WT/ND mice and EPA supplementation did not change them. By contrast, serum adiponectin levels in *Insr*^*P1195L/+*^/HFD mice were significantly lower than those in *Insr*^*P1195L/+*^/ND mice. In addition, EPA supplementation significantly increased serum adiponectin levels in *Insr*^*P1195L/+*^/HFD mice. We then evaluated correlation between body weight and adiponectin levels (Figure 5b) and between blood glucose levels and adiponectin levels (Figure 5c). Interestingly, we found the negative correlation between body weight and adiponectin levels in *Insr*^*P1195L/+*^ mice (*r*=−0.502, *P*<0.001) (*n*=76) but not in WT (*n*=70) mice. EPA supplementation decreased body weight and increased adiponectin levels in *Insr*^*P1195L/+*^/HFD mice. These data suggest that *Insr*^*P1195L/+*^ mice are susceptible to hypoadioponectinemia in response to obesity. Furthermore, there was a clear negative correlation between blood glucose levels and adiponectin levels in all the experimental groups, including WT and *Insr*^*P1195L/+*^ mice (*r*=−0.628, *P*<0.001) (*n*=146). EPA supplementation increased adiponectin levels proportionally to the decrease in blood glucose levels in *Insr*^*P1195L/+*^/HFD mice, suggesting that EPA supplementation improves hyperglycemia through the action of adiponectin.

To investigate the mechanism of the increase of serum adiponectin levels by EPA, mRNA expression of adiponectin was quantified. The mRNA expression levels of adiponectin were significantly increased by EPA supplementation in *Insr*^*P1195L/+*^/HFD mice (Figure 5d). Interestingly, mRNA expression of *Pparg2*, which is known to be transactivated by insulin,^14^ also was changed by EPA with a trend similar to that of adiponectin (*Acrp30*) mRNA (Figure 5e).

## Discussion

EPA is included in fish oil and there is a lot of epidemiological and experimental data showing that EPA has an inhibitory effect on cardiovascular diseases.^15^^,^^16^ In addition, EPA has been shown to decrease serum TG levels^17^ and inhibit platelet aggregation.^18^ However, the antidiabetic effect of EPA is still controversial.^19^

Our present results show that EPA treatment significantly improved blood glucose levels, glucose tolerance and insulin sensitivity in *Insr*^*P1195L/+*^/HFD mice. We recently reported that HFD feeding induces hyperglycemia in *Insr*^*P1195L/+*^/HFD mice through increased lipolysis and increased gluconeogenesis from glycerol.^3^ Interestingly, abnormal upregulation of *G6pc*, the critical regulator of gluconeogenesis, was significantly ameliorated in the *Insr*^*P1195L/+*^/HFD liver by EPA supplementation. By contrast, EPA supplementation did not affect *G6pc* expression in the WT/HFD liver in accordance with the previous report in cafeteria diet-fed Wistar rats.^20^ Furthermore, EPA supplementation suppressed the increase in blood glucose levels after glycerol loading, which was shown to increase *G6pc* in the liver.^3^ These results suggest that EPA elicits its antidiabetic effect through the suppressed gluconeogenesis from glycerol. Considering that insulin suppresses *G6pc* expression in the liver, we examined whether EPA improves insulin resistance through its direct action on hepatocytes using primary hepatocytes from WT/HFD and *Insr*^*P1195L/+*^/HFD mice. Our present studies in primary hepatocytes suggest that EPA might improve insulin sensitivity through a PI3K-independent pathway in *Insr*^*P1195L/+*^/HFD mice.

We then evaluated the effect of EPA on lipid metabolism in WAT. EPA has been reported to suppress chronic inflammation in WAT^21^ through various pathways, including activation of GPR120 and inhibition of Toll-like receptor 4.^22^ Oh *et al.*^7^ reported that *n*-3 PUFA lessens insulin resistance through a signal involving GPR120 expressed in adipocytes and adipose tissue macrophages. Considering that EPA supplementation reduced *Tnfa* and *Emr1* expression levels in WAT and serum FFA levels, anti-inflammatory action of EPA may contribute to the amelioration of hyperglycemia, at least in part, in *Insr*^*P1195L/+*^/HFD mice.

Interestingly, EPA supplementation reduced adiposity in both WT/HFD and *Insr*^*P1195L/+*^/HFD mice. Adiposity is known to influence release of adipocytokines from WAT to mediate insulin sensitivity.^23^ Especially, adiponectin has been shown to improve insulin sensitivity in various insulin target tissues, such as liver and skeletal muscle,^24^ and to be decreased in obesity and T2DM.^13^ Nevertheless, *Insr*^*P1195L/+*^/HFD mice exhibited lower adiponectin levels compared with those of WT/HFD mice despite the lower body weight. Although no correlation between body weight and adiponectin levels was detected in WT mice, there was a large decrease in adiponectin levels in response to body weight gain by HFD in *Insr*^*P1195L/+*^ mice. These results suggest that *Insr*^*P1195L/+*^ mice are susceptible to hypoadiponectinemia in response to body weight gain by HFD. By contrast, there was a clear negative correlation between blood glucose levels and adiponectin levels in *Insr*^*P1195L/+*^ mice. Notably, EPA supplementation improved hyperglycemia of *Insr*^*P1195L/+*^/HFD mice along with the increase in adiponectin levels. In this study, a causal relationship between hypoadiponectinemia and hyperglycemia has not been shown. However, EPA supplementation increases mRNA expression of *Pparg2*, which has been reported to be transactivated through insulin.^14^ In addition, Oh *et al.*^7^ has reported that *n*-3 PUFA potentiates insulin signaling through Akt phosphorylation in adipocytes. Accordingly, EPA might improve insulin signaling in adipocytes of *Insr*^*P1195L/+*^/HFD mice, which could ameliorate their hypoadiponectinemia. Adiponectin has been reported to suppress gluconeogenesis through both AMPK signaling-dependent and -independent pathways.^24, 25^ Accordingly, in the *Insr*^*P1195L/+*^/HFD liver, decreased adiponectin levels could elicit the increase in gluconeogenesis in the liver and lead to the development of hyperglycemia. Taken together, EPA supplementation is considered to act directly on hepatocytes to improve insulin signaling and indirectly to improve insulin signaling through enhancement of adiponectin secretion from WAT. In large clinical studies, EPA has been reported not to show an antidiabetic effect in human T2DM patients.^26^ However, the pathophysiology of T2DM is known to be heterogeneous. Our present study suggests that EPA supplementation may be effective in the treatment of T2DM patients with a pathophysiology similar to that of *Insr*^*P1195L/+*^/HFD mice. Thus, considering personalized medicine to various T2DM patients, EPA supplementation might be one of the choices of therapeutics.
